# Comparative Study of Oxidative Stress Responses in Pediatric Type 1 Diabetes and Transient Hyperglycemia

**DOI:** 10.3390/ijms26041701

**Published:** 2025-02-17

**Authors:** Anca Daniela Pinzaru, Ancuta Lupu, Tatiana Chisnoiu, Ginel Baciu, Alexandru Paul Baciu, Carmen Baciu, Vasile Valeriu Lupu, Adriana Luminita Balasa, Sergiu Chirila, Florin Gabriel Panculescu, Doina Catrinoiu, Simona Claudia Cambrea, Ramona Mihaela Stoicescu, Cristina Maria Mihai

**Affiliations:** 1 Pediatrics, County Clinical Emergency Hospital of Constanta, 900591 Constanta, Romania; pinzaruancadaniela@gmail.com (A.D.P.); adriana.balasa@365.univ-ovidius.ro (A.L.B.); cristina_mihai@365.univ-ovidius.ro (C.M.M.); 2 Department of Pediatrics, Faculty of Medicine, “Ovidius” University, 900470 Constanta, Romania; 3 Department of Pediatrics, “Grigore T. Popa” University of Medicine and Pharmacy, 700115 Iasi, Romania; ancuta.ignat1@umfiasi.ro (A.L.); vasile.lupu@umfiasi.ro (V.V.L.); 4 Department of Pediatrics, “Dunărea de Jos” University of Galati, 800008 Galati, Romania; baciu91@yahoo.com; 5 Hyperclinic Medlife Galati, 800150 Galati, Romania; carmengavrilaa@gmail.com; 6 Faculty of Medicine, “Ovidius” University, 900470 Constanta, Romania; sergiu.chirila@univ-ovidius.ro (S.C.); gabriel.panculescu@yahoo.ro (F.G.P.); dcatrinoiu@gmail.com (D.C.); 7 Department of Infectious Diseases, Faculty of Medicine, “Ovidius” University, 900470 Constanta, Romania; claudia.cambrea@365.univ-ovidius.ro; 8 Department of Microbiology and Immunology, Faculty of Pharmacy, “Ovidius” University of Constanta, Str. Căpitan Aviator Al. Șerbănescu, nr.6, Campus Corp C, 900470 Constanta, Romania; ramona.stoicescu@365.univ-ovidius.ro

**Keywords:** diabetes, children, oxidative stress, transient hyperglycemia

## Abstract

Hyperglycemia significantly initiates oxidative stress in children diagnosed with type 1 diabetes (T1DM). This study investigates the differences in oxidative stress markers between pediatric patients with T1DM and those experiencing transient hyperglycemia. In this case–control study, 42 children diagnosed with T1DM, according to ISPAD (International Society for Pediatric and Adolescent Diabetes), and their healthy counterparts, aged 1–6 years old, participated. Blood samples were analyzed for oxidative stress biomarkers such as malondialdehyde (MDA) and glutathione peroxidase (GPx). There was no statistically significant association found between the A1c % and age, BMI, and insulin dose (*p* > 0.05). A negative correlation was found between Se, Zn, cholesterol, GSH, and GPx (*p* < 0.05), as well as a statistically meaningful positive correlation with the A1c % (*p* < 0.001). GSH exhibited a statistically significant negative correlation (*p* < 0.001) with diabetic group. In comparison to control participants, plasma MDA levels (1.3 ± 0.36 µmol/L) had already increased significantly. MDA did correlate in a diabetic group with triglyceride levels (*p* > 0.0001) or total cholesterol. In the healthy group, the cholesterol levels were normal and apparently did not influence MDA levels. The oxidative state remained unchanged in the healthy participants experiencing temporary hyperglycemia, even though T1DM altered the link between selenium, zinc, and lipids.

## 1. Introduction

Type 1 diabetes (T1DM) is an autoimmune disease characterized by hyperglycemia secondary to pancreatic beta cell destruction leading to insufficient insulin production. Maintaining normal blood sugar and avoiding hypoglycemia or hyperglycemia is achieved by administering the treatment (insulin) after evaluating all factors (type of insulin, blood sugar level, other associated chronic or acute diseases, stress, and hormonal imbalances) [1]. These steps are difficult to achieve and maintain in the absence of adequate medical education. Education targets both the patient and their family. In this regard, international forums have published guidelines for treatment and dietary recommendations in order to maintain a balanced life [2].

Maintaining appropriate glycemic management is a crucial factor in mitigating the advancement of diabetes complications, which impacts the antioxidant defense (AOD) system [2]. The human body has evolved a defensive mechanism to counteract the harmful effects of oxygen metabolism by employing enzymatic and non-enzymatic systems to combat reactive oxygen species (ROS). The process mentioned above depends on the interaction between certain enzymes, such as superoxide dismutase (SOD) or glutathione peroxidase (GPx), to counteract ROS and so safeguard crucial cellular components [2].

Romania shows an upward trend in the spread of diabetes among children. According to Vlad et al., after a plateau, the incidence of T1DM showed an exponential increase during the COVID-19 pandemic [3].

T1DM presents major complications due to uncompliant patients, with an important impact on the social and economic stability of every society. Implementing a strict and proper glycemic balance reduces the risk of short- (hypoglycemia) and long-term effects (kidney failure, neurological deficiencies, vision impairment, circulation problems) on the patient and on the economy. A proper blood glucose level reduces the progression of diabetes-related complications in children and adolescents with T1DM. Maintaining diabetes control is the main focus for every medical team and patient [4].

Optimal diabetes management is achieved through intensive glycemic control, which includes frequent blood glucose monitoring using sensors, accurate insulin administration via injections or insulin pumps, and adherence to a structured regimen of diet and physical activity [3].

The involvement of the immune system in the pathogenesis of T1DM is intensely analyzed by researchers, including the entire disease progression from its onset to the eventual destruction of β cells [5,6]. Several investigations have demonstrated that oxidative stress (OS) is associated with the deterioration of pancreatic islets, resulting in the demise of β cells by either necrosis or apoptosis [5,7]. Diabetic children are subject to heightened levels of OS due to many mechanisms, such as glucose autooxidation and non-enzymatic protein glycation [4]. Under typical physiological circumstances, organisms maintain a crucial equilibrium between the production of oxygen free radicals and the utilization of AOD mechanisms. These defense systems serve to neutralize and safeguard against the harmful effects of free radicals [6].

The presence of an imbalance between oxidants and antioxidants gives rise to a physiological state commonly referred to as oxidative stress. Several mechanisms contribute to OS in T1DM, including glucose autooxidation, non-enzymatic protein glycation, and the activation of pathways such as nicotinamide adenine dinucleotide phosphate (NADPH)-dependent aldose reductase [8,9,10]. Nonenzymatic glycation refers to an inherent chemical process that occurs spontaneously when glucose reacts with the amino groups present in proteins. This reaction leads to the formation of reversible Shiff bases initially, which subsequently transform into more stable Amadori products [11]. These processes can reduce glutathione levels and promote the formation of advanced glycation end products, which are implicated in long-term diabetic complications. Studies have shown that OS not only contributes to pancreatic β-cell apoptosis, but also plays a role in the development of microvascular and macrovascular complications associated with diabetes [12].

The purpose of the study is to demonstrate the importance of education in the management of hyperglycemia. Starting from the idea that hyperglycemia is the key element in triggering oxidative stress, the aim of the study was to observe whether healthy children show the same molecular changes as those with chronic hyperglycemia. This would imply an increased risk of developing microvascular complications even from childhood. When these components are shown, we can modify the therapy approach for healthy children with isolated hyperglycemia, potentially altering their future health trajectory.

Isolated hyperglycemia does not constitute a substrate for triggering or maintaining oxidative stress. In a diabetic child, maintaining good metabolic control is the key to avoiding the long-term complications characteristic of diabetes.

## 2. Results

The study sample comprised 42 T1DM children, with equal sex distribution, with a mean age of 4.3 ± 1.5 and a BMI of 17.3 ± 0.2. The control group consisted of 42 healthy children, with a mean age of 5.4 ± 1.2, 50% male and 50% female, and a BMI of 17.6 ± 0.7 (Table 1).

The mean age of the diabetic group was 4.3 years, and the duration of illness varied from 1 to 11 years. The insulin dose at onset was between 0.2 and 1.0 units/kg; 45% of the patients used basal-bolus, and 45% underwent continuous blood glucose monitoring and insulin infusion with one type of insulin. There were no discernible changes in either group’s age, sex or BMI (Table 1). Compared to the control group, the FPG was statistically substantially more significant in diabetic patients.

There was no statistically significant association found between age and BMI. A statistically negative correlation was found between Se, Zn and GSH, but a statistically meaningful positive correlation was found with the A1c % (*p* < 0.001). GSH exhibited a statistically significant negative correlation (*p* < 0.001) with diabetic group (Table 2).

**Figure 1:**
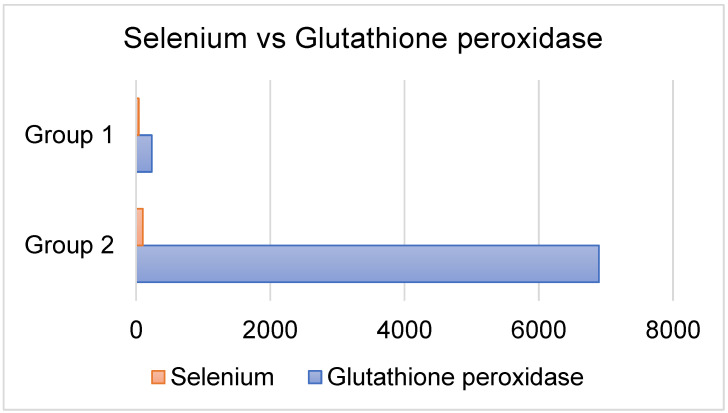
Correlation between Se and GSH (negative correlation). Group 1—T1DM group. Group 2—Control group.

In comparison (Figure 1) to control patients (6897 ± 53.12 U/L; *p* < 0.01), erythrocyte GPx activity was considerably reduced in diabetic subjects (235 ± 98 U/L). Nevertheless, in patients with diabetes, there was a positive correlation between MDA and total cholesterol (*p* < 0.01).

In comparison to control participants (0.56 ± 0.023 µmol/L; *p* < 0.0001), plasma MDA levels (1.3 ± 0.36 µmol/L) had already increased significantly (Table 3). MDA did correlate in a diabetic group with triglyceride levels (*p* > 0.0001) or total cholesterol. In the healthy group, the cholesterol levels were normal and apparently did not influence MDA levels (Table 3). Although no statistically significant relationship was found between MDA and triglyceride levels in the diabetic group, a trend suggesting a possible correlation was observed

**Figure 2:**
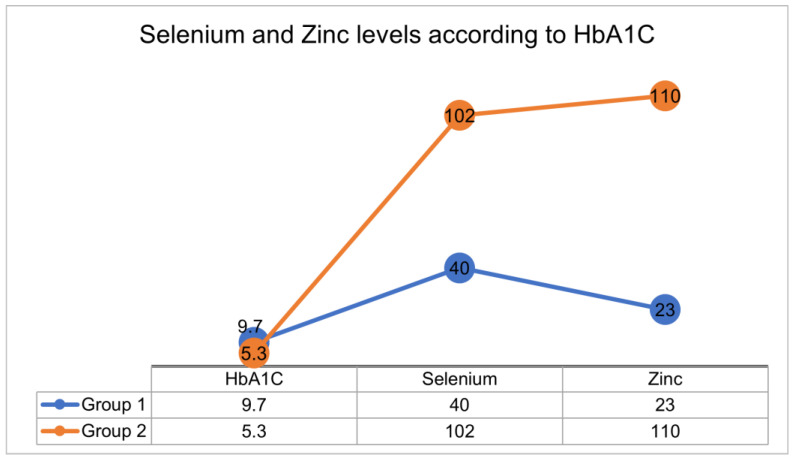
Se and Zn levels according to HbA1C.

In comparison to their healthy peers (Figure 2), adolescents with T1DM exhibited lower levels of Zn (23.23 μg/dL vs. 110 μg/dL, *p* < 0.001)

In the current investigation (Table 4), we did not find any significant associations between the degree of acidosis upon diagnosis (pH and bicarbonate values), peroxidation parameters (MDA), or any of the antioxidants evaluated (GPx, zinc, selenium) in the plasma of diabetic children 9–10 days after the clinical onset of diabetes. Age, sex, or variations in GPx did not affect OS markers or antioxidant levels in plasma in the control group.

Values are reported as the median or as the subjects’ percentage (%). Using Kruskal–Wallis ANOVA tests with post hoc analysis, statistically significant differences between the medians were found. The following terms and acronyms are used in this context: total antioxidant status (TAS), total oxidant status (TOS), zinc (Zn), non-significant (NS), OS, and AOD.

## 3. Discussion

Our research, which indicates that the serum levels of zinc and selenium in the diabetic group were significantly lower than those in the control group, underscores the need for further research in this area. This aligns with the findings of Ahmed et al., who identified that children with T1DM exhibited lower serum levels of Se and Zn than the control group [13]. Grabia et al. compared 105 T1DM patients with 65 healthy children, obtaining similar results. During diabetes onset, the levels of selenium and zinc tend to drop drastically due to hyperglycemia effects on the OS [14]. Another possible explanation for the decrease in selenium levels can be offered by the increase in selenium consumption secondary to the increase in the action of GPx, which reduces the level of free radicals created by elevated OS, as Özenç et al. explained in his research [15].

The relationship between zinc, T1DM, and OS has gained attention due to zinc’s role in the insulin complex. This has led some experts to propose a potential correlation between diabetes and zinc deficiency [16,17]. However, none of the patients in our study exhibited zinc deficiency, in contrast to what is observed with other essential elements. Zinc is not stored in significant amounts in the human body, so inadequate dietary intake can contribute to complications, particularly regarding OS in T1DM [18,19]. Despite this, there is limited research specifically addressing children and adolescents with T1DM, and the results from these studies remain inconsistent [17,19]. Our findings align with previous studies that found no significant difference in serum zinc levels between children and adolescents with T1DM and their healthy peers. However, it is worth noting that other studies have reported varying levels of serum zinc in children with T1DM, with some showing lower levels [17,19] and others showing higher levels [20]. These results suggest that while zinc may not exhibit a clear deficiency in children with T1DM, fluctuations in its levels still warrant further investigation in the context of oxidative stress.

Zinc and selenium both play crucial roles in managing oxidative stress, particularly in the context of T1DM. Zinc is an essential component of the insulin complex, and its potential deficiency has been linked to diabetic complications due to its involvement in OS processes [16,17]. However, none of the participants in our study exhibited zinc deficiency, aligning with findings from previous research that reported no significant differences in serum zinc levels between children and adolescents with T1DM and healthy controls. This suggests that while zinc may not show a clear deficiency in this population, its fluctuating levels still warrant further study. Indeed, studies have reported inconsistent serum zinc levels in T1DM, with some indicating lower levels [17,19], and others higher levels [20].

Similarly, selenium, an integral component of the enzyme GPx, plays a significant role in reducing OS by converting hydrogen and organic peroxides into less harmful substances such as alcohol and water [21]. Seleno-methionine, the primary reservoir of selenium, is not produced endogenously, which means it must be obtained through dietary sources or supplementation. Selenium’s function in reducing lipid peroxidation and mitigating ROS further underscores its importance in managing OS in T1DM [21]. Our study corroborates these findings, as we observed no significant deviations in selenium levels among participants with T1DM, suggesting that while supplementation or dietary intake may be necessary for optimal selenium status, the relationship between selenium and OS in T1DM remains complex and requires additional investigation. Selenium in pediatric research remains incompletely studied. Nie et al. analyzed over 4000 adults with diabetes. In comparison to healthy individuals, diabetic patients had notably reduced levels of serum selenium, indicating that individuals with diabetes may possess heightened metabolic requirements for selenium [22]. In the present investigation, it was observed that there were discernible disparities in selenium concentrations between preschoolers diagnosed with T1DM and their unaffected counterparts, similar to data obtained by Salmonowicz et al. after analyzing 87 children with diabetes and 41 healthy peers [14,23]. Selenium exhibits a notable capacity as an antioxidant, influencing the overall antioxidant equilibrium inside the human body [23]. GPx represents a highly significant selenoenzyme. The principal objective of this function is to facilitate the catalytic conversion of hydrogen peroxide into water [14,24]. Darenskaya observed a reduction in GPx activity in individuals with diabetes, a finding that aligns with the results of our current investigation. Furthermore, the observed percentage was approximately 20% lower in individuals with a prolonged duration of T1DM than in the early onset group. The observed outcome could lead to a diminished amount of glutathione or the deactivation of the enzyme due to heightened OS [25].

The present study investigated the activity of GPx in red blood cells, revealing a statistically significant decrease in diabetic patient compared to control participants. Dominguez et al. conducted a study that yielded the same findings, demonstrating that individuals diagnosed with T1DM exhibited lower GPx activity than their healthy counterparts. The level of GPx in children with diabetes tends to decrease compared to their siblings and parents [26]. Darmauan et al. wanted to evaluate the structure of GPx in the diabetic child, establishing step by step the moment of the installation of the deficiency. The current study indicates that blood glutathione levels are notably reduced in adolescents diagnosed with uncomplicated T1DM compared to a group of healthy volunteers matched in age and sex. Furthermore, this reduction in glutathione levels is due to a decline in the synthesizing rate. It occurs regardless of whether the rate of glutathione fractional synthesis remains unchanged or increases, depending on the level of blood glucose control [27].

Previous research has consistently shown that individuals with diabetes exhibit elevated levels of MDA in their serum compared to individuals without diabetes, a finding that is consistent with our study [28,29]. In line with earlier studies, our investigation also demonstrated that the lipid profile of individuals with T1DM was notably lower than that of the healthy control group. Furthermore, the levels of MDA, a key biomarker for assessing lipid peroxidation and OS, were significantly elevated in the T1DM group compared to control group. These findings align with research suggesting that elevated blood glucose levels in diabetes contribute to increased lipid peroxidation and reduced AOD, thereby exacerbating OS [29]. The significantly elevated levels of MDA observed in children with T1DM compared to those with transient hyperglycemia and the control group confirm previous studies linking oxidative damage to chronic hyperglycemia and impaired AOD mechanisms [28]. These findings reinforce the hypothesis that persistent hyperglycemia exacerbates lipid peroxidation, contributing to long-term diabetes complications.

Hyperglycemia, whether transient or persistent as in T1DM, is associated with increased oxidative stress, which is a key contributor to cellular damage [30]. The pathogenesis of OS in T1DM is described in this guideline, along with methods for management [31]. Children possess more efficient AOD mechanisms, including higher levels of endogenous antioxidants such as SOD, catalase (CAT), and GPx [23]. These enzymes help mitigate oxidative stress, potentially preventing significant alterations in oxidative markers during brief hyperglycemic episodes.

In T1DM, chronic hyperglycemia leads to sustained oxidative stress, while in transient hyperglycemia, the OS may be temporary but significant, facts that were demonstrated in a comprehensive review of OS mechanisms in pediatric conditions [32]. Bunnag et al. provided a comparative analysis of oxidative markers in transient hyperglycemia and T1DM [33]. Ceriello et al. explored the role of glycemic variability in driving OS in diabetes [34]. ROS-mediated endothelial damage promotes the activation of inflammatory pathways, increases vascular permeability, and disrupts nitric oxide (NO) bioavailability, leading to impaired vasodilation [35]. This mechanism is central to the pathogenesis of diabetic nephropathy, retinopathy, and neuropathy. Additionally, OS triggers the activation of pro-apoptotic signaling pathways, including the JNK and NF-κB pathways, which further exacerbate tissue damage and inflammation in diabetes [36]. OS plays a critical role in endothelial dysfunction, which is a key contributor to diabetic microvascular and macrovascular complications [37]. Chronic hyperglycemia leads to the excessive production of ROS through pathways such as glucose auto-oxidation, the activation of the polyol pathway, the increased formation of advanced glycation end-products (AGEs), and mitochondrial dysfunction [38]. These ROS induce oxidative damage in lipids, proteins, and DNA, impairing cellular homeostasis and leading to β-cell dysfunction [39].

However, transient hyperglycemia may not sustain these pathways long enough to induce measurable oxidative damage [36,40]. Furthermore, studies indicate that age-related differences in glucose metabolism could contribute to this phenomenon. Younger children have higher insulin sensitivity and a greater ability to utilize glucose efficiently, reducing OS accumulation compared to adolescents and adults [39].

The findings of this study provide insights into how OS mechanisms differ between these two conditions in pediatric patients, emphasizing the importance of early detection and management strategies. While hyperglycemia is a key driver of oxidative stress, the study suggests other contributing factors unique to T1DM, such as chronic inflammation and autoimmune processes [41]. Pro-inflammatory cytokines, common in T1DM, may exacerbate OS independently of glucose levels [42]. In transient hyperglycemia, the absence of such chronic inflammatory stimuli likely accounts for the transient nature of OS [41,42]. OS is exacerbated by the interaction of autoimmune and inflammatory pathways in T1DM, which leads to beta-cell death and the progression of the illness [43].

Recent studies have explored therapeutic strategies targeting OS in T1DM [44,45]. Natural antioxidants, such as polyphenols and vitamins, and pharmacological agents like metformin have shown potential in mitigating OS and protecting beta cells [46]. Additionally, emerging gene-editing technologies like CRISPR/Cas9 are being investigated for restoring beta-cell function by targeting redox-related pathways [40]. Another review highlights the mechanisms by which autoimmune responses intensify oxidative damage and beta-cell dysfunction, underscoring their therapeutic potential in targeting these pathways [47].

Oxidative stress-mediated mitochondrial dysfunction is a shared hallmark of asthma and T1DM. In asthma, mitochondrial damage impairs airway cell repair, while in T1DM, it compromises insulin secretion and beta-cell survival [48].

One important physiopathogenic mechanism in childhood obesity has been identified as the imbalance between antioxidants and OS [49]. Cellular damage results from this imbalance, which happens when the body’s AODs are outpaced by the generation of ROS [50]. Through hyperglycemia, hyperlipidemia, excessive caloric intake, and chronic inflammation, childhood obesity exacerbates oxidative stress. The development of metabolic syndrome and related comorbidities including insulin resistance and cardiovascular illnesses is influenced by these variables, which lead to elevated lipid peroxidation, protein carbonylation, and mitochondrial dysfunction [51,52]. Recent advancements focus on using novel pharmacological agents, such as GLP-1 receptor agonists, which not only promote weight loss but also appear to reduce OS by improving metabolic profiles [52]. Moreover, lifestyle interventions like diet modifications rich in antioxidants (fruits, vegetables, omega-3 fatty acids) and increased physical activity remain critical in managing OS in this population [53].

With variations in its manifestation and effects on the course of the disease, OS is a key component of the pathophysiology of both pediatric T1DM and cystic fibrosis (CF). Given that OS is linked to inflammatory pathways, organ damage, and metabolic problems, this is especially important when comparing these two disorders and their associations with temporary hyperglycemia [54]. To prevent metabolic complications, continuous glucose monitoring (CGM) has proven to be an effective tool, especially in pediatric patients with T1DM, offering a more accurate and immediate picture of glycemic trends compared to traditional finger-stick tests [55].

In the pathophysiology of T1DM and chronic hepatitis, OS plays a crucial role. Both conditions are characterized by persistent inflammation and immunological activity, which contribute to the overproduction of ROS [55]. These ROS cause damage to cellular structures, including lipids, proteins, and DNA, leading to cellular dysfunction and accelerated disease progression. In T1DM, chronic hyperglycemia exacerbates oxidative stress, creating a feedback loop that further enhances inflammation and immune activation. Similarly, in chronic hepatitis, prolonged viral infection or autoimmune responses trigger continuous inflammatory processes, which also promote ROS generation [56]. This sustained oxidative damage can impair organ function and contribute to the development of complications such as vascular damage, insulin resistance, and liver fibrosis. The accumulation of ROS in both conditions highlights the importance of AOD mechanisms, which can mitigate some of the oxidative damage and potentially slow disease progression [56].

Many variables that affect the severity and consequences of the illness, particularly in those with pre-existing disorders like diabetes, have come to light as a result of COVID-19 [57]. OS is a key factor in the pathophysiology of both diabetes and COVID-19, according to research. When the two diseases coexist, the body frequently faces additional difficulties that worsen tissue damage, inflammation, and immunological dysfunction [58].

The study provides a comparative analysis of OS markers in T1DM and transient hyperglycemia, highlighting key differences in their pathophysiology. The pediatric focus offers valuable insights into early-stage disease processes.

## 4. Materials and Methods

This case–control study included 42 children diagnosed with T1DM according to the criteria of the International Society for Pediatric and Adolescent Diabetes (ISPAD) [59], as well as 42 age-matched healthy counterparts. The study was conducted between January 2019 and January 2023 at the Department of Pediatric Diabetology, Department of Pediatrics, Clinical County Hospital “Sf. Apostol Andrei”, Constanța.

The diabetic group inclusion criteria are as follows:

- Diagnosis of T1DM according to ISPAD criteria;
- Presence of autoimmune markers (islet autoantibodies);
- Absence of other underlying metabolic or autoimmune diseases (e.g., renal disease, celiac disease, thyroid dysfunction).

The control group selection and criteria are as follow:

- Children of the same age range (1–6 years) who were admitted for isolated hyperglycemia, with no diagnosis of diabetes or other metabolic conditions;
- Screening for prediabetes and metabolic disorders was performed before inclusion;
- After comprehensive clinical and biological assessment, diabetes was excluded, and the diagnosis of isolated hyperglycemia was confirmed, requiring dynamic monitoring.

All participants underwent detailed anthropometric and biochemical evaluations, including the following:

- Anthropometry—Body mass index (BMI) assessment (weight/height ratio);
- Biochemical tests—Mean glucose level, HbA1c, cholesterol and its derivatives, triglycerides, autoimmune markers, and urine analysis.

Each patient and caregiver completed a questionnaire regarding dietary habits, medical history, insulin therapy, and general health information. The nutritional interview provided insights into the dietary intake of substances with antioxidant potential.

Blood samples were collected in heparin and EDTA tubes. All samples were processed within one hour of collection to ensure reliability. The specific techniques employed for each marker are as follows:

- Malondialdehyde (MDA)—Measured using high-performance liquid chromatography (HPLC) with fluorescence detection, a highly sensitive and specific method for detecting lipid peroxidation products. This method involves the derivatization of MDA with thiobarbituric acid (TBA) to form a fluorescent adduct, which is then separated and quantified using HPLC;
- Glutathione peroxidase (GPx)—Determined using a photometric assay that measures enzymatic activity in erythrocytes. This method relies on the oxidation of glutathione (GSH) to glutathione disulfide (GSSG) by GPx in the presence of cumene hydroperoxide. The rate of NADPH oxidation, catalyzed by glutathione reductase, is monitored at 340 nm to determine GPx activity;
- Zinc (Zn)—Quantified using inductively coupled plasma mass spectrometry (ICP-MS), a highly precise and sensitive technique that allows for the detection of trace metal concentrations in plasma;
- Selenium (Se)—Measured using ICP-MS, ensuring the accurate quantification of selenium levels in plasma, given its importance in AOD systems, particularly as a cofactor for GPx.

Informed consent was obtained from all participants and their legal guardians after reviewing the study details. The study was approved by the Ethics Committee of the Clinical County Hospital “Sf. Apostol Andrei”, Constanța.

Following collection, it is crucial that sample processing is conducted in strict accordance with the laboratory’s guidelines. This ensures the integrity and reliability of the results, providing a secure foundation for our research.

Plasma must be isolated using centrifugation for 15 min at 1500× *g* [g = (1118 + 10^−8^) × (radius in cm) × (rpm)^2^]. A plastic pipette was utilized to isolate the plasma. If this was not feasible, the plasma was preserved at –20 °C in a plastic container (polypropylene or polystyrene). Plasma samples collected externally were transported to a container designated for frozen samples.

The monitoring protocol was structured to comply with ISPAD guidelines for evaluating the metabolic rate in pediatric patients. This commitment to high standards ensured the quality and reliability of our research, providing reassurance to our colleagues and collaborators.

The quantification of SOD, MDA, and GPx in the blood was conducted by obtaining venous blood in a vacutainer containing EDTA using the photometric/enzymatic method.

The cut-off values for antioxidant capacity were as follows: low antioxidant capacity—<280 µmol/L, average antioxidant capacity—280–320 µmol/L, increased antioxidant capacity—>320 µmol/L. The oxidative capacity cut-offs were as follows: low—110–120 μg/L, average—150–175 μg/L, high—above 198 μg/L. The cut-off values for selenium were as follows: 40–160 μg/L. Values < 40 μg/L usually indicate a selenium deficiency associated with the loss of GPx activity. The cut-off for zinc was 44–115 μg/dL

Complete urinalysis includes the physical (color, appearance, density), chemical (pH, proteins, glucose, ketone bodies, bilirubin, urobilinogen, nitrites, leukocyte esterase activity, hemoglobin), and microscopic examination of urine sediment.

Statistical program (The R program, version 4.2.3 Copyright (C) 2023) was used to perform a statistical analysis on the data. For the statistical study, the R Foundation for Statistical Computing, R Core Team (2023) was consulted. This is a language and environment for computing statistics. Vienna, Austria’s R Foundation for Statistical Computing (https://www.R-project.org, accessed on 30 January 2023) was used. The Kruskal–Wallis ANOVA test with post hoc analysis and the Mann–Whitney U test were used for quantitative variables. A scree plot was used to ascertain the number of dimensions that could accurately represent the data; the data were then examined and shown as a Burt matrix. The usefulness of OS and AOD indicators as diagnostic tools was evaluated using receiver operating characteristic (ROC) curve analysis.

## 5. Conclusions

In conclusion, a positive association was observed between GPx activity, zinc levels, and blood selenium concentrations. Despite the presence of temporary hyperglycemia, the oxidative state remained stable in healthy participants. However, in individuals with T1DM, the relationship between selenium, zinc, and lipid levels was altered, indicating potential changes in oxidative processes. While the study provides valuable insights, the relatively small sample size limits the generalizability of these findings. Furthermore, the lack of longitudinal data restricts our ability to draw conclusions regarding the long-term effects of transient hyperglycemia-induced oxidative stress. Additional research with larger sample sizes and longer follow-up is needed to confirm these findings and elucidate the underlying mechanisms.

## Figures and Tables

**Table 1:** Division of the analyzed group according to their metabolic control.

Parameter	Group 1 (T1DM) (n = 42)	Group 2 (Control Group) (n = 42)
Age (years) BMI (Kg/m^2^) Gender (girls/boys)	4.3 ± 1.5	5.4 ± 1.2
	17.3 ± 0.2	17.6 ± 0.7
	G vs. B: 21/21	G vs. B: 21/21
T1DM duration (years) Type of insulin therapy	Onset	-
	45% of the patients used basal-bolus, and 45% underwent continuous blood glucose monitoring and insulin infusion with one type of insulin.	-
HbA1c (%)	9.7	5.3
Type of glucose monitoring system Glucose mean levels mg/dL	45% CII, 45% CGM, 45% MDI	Capillary testing for glucose levels at least 5–7 times/day
	356 +/− 125	186 +/− 53

Note—The median or percentage of subjects (%) is used to express values. Abbreviations include the following: multiple daily injections (MDI); continuous glucose monitoring (CGM); continuous subcutaneous insulin infusion (CSII); glycated hemoglobin (HbA1c); type 1 diabetic mellitus (T1DM).

**Table 2:** Laboratory presentation of analyzed groups.

	Group 1 (T1DM)	Group 2 (Control Group)	
Total cholesterol HDL cholesterol LDL cholesterol	265.73 +/− 63.21	102.06 +/− 22.2	≤0.05 *
	22.31 +/− 5.1	56.23 +/− 15.22	≤0.05 *
	151 +/− 73.8	106.21 +/− 12.3	≤0.05 *
Triglyceride Malondialdehyde µmol/L	123 +/− 23.25	90.5 +/− 25.23	≤0.05 *
	1.3 +/− 0.36	0.56 +/− 0.023	<0.001 **
Glutathione peroxidase U/L Selenium µg/L	235 +/− 98	6897 +/− 53.12	<0.001 **
	40 +/− 0.25	102 +/− 2.5	<0.001 **

Note: * significant; ** highly significant.

**Table 3:** Division of the analyzed group according to their antioxidant status and metabolic control.

	Diabetic Group (n = 42)	Control Group (n = 42)	*t*-Test
Zn (μg/dL) mean ± SD Se (ng/mL) mean ± SD	23.23 +/− 0.56 μg/dL	110 +/− 0.25 μg/dL	2.00
	40 +/− 0.25	102 +/− 2.5	9.5
Malondialdehyde µmol/L	1.3 +/− 0.36	0.56 +/− 0.023	6.2
GPx (U/L) mean ± SD A1c % mean ± SD	1.69 ± 0.6 (0.8–2.8)	2.74 ± 0.46 (2.0–3.6)	7.0
	9.7 ± 1.6	5.3 +/− 0.6	8.7

Note: A1c—glycosylated hemoglobin; GPx—glutathione peroxidase; Se—selenium; Zn—zinc.

**Table 4:** Glycemia levels and the percentage of individuals with and without T1DM classified into each group according to the level of AOD and OS parameter (TAS, TOS and Zn and Se).

Parameter	Low		High		*p*-Value	
	%		%		Low vs. Medium	Low vs. High
TAS	23	8.3 (6.4–12.6)	25	6.9 (6.3–8.7)	NS	<0.05
TOS	15	8.8 (5.3–9.9)	51	10.8 (9.0–12.5)	<0.01	<0.01
Se	55	7.5 (6.6–9.8)	20	8.2 (7.3–9.8)	NS	NS
Zn	13	7.5 (6.73–10.5)	52	8.1. (6.7–9.9)	NS	NS

## Data Availability

The data are available on request from the corresponding author.

## Abbreviations

The following abbreviations are used in this manuscript:

AOD	Antioxidant defense
ROS	Reactive oxygen species
SOD	Superoxide dismutase
GPx	Glutathione peroxidase
MDA	Malondialdehyde
NADPH	Nicotinamide adenine dinucleotide phosphate
ISPAD	International Society for Pediatric and Adolescent Diabetes
HbA1c	Glycosylated hemoglobin
BMI	Body mass index
MDI	Multiple daily injections
CGM	Continuous glucose monitoring
CSII	Continuous subcutaneous insulin infusion
FPS	Fasting plasma glucose
Se	Selenium
Zn	Zinc
TAS	Total antioxidant status
TOS	Total oxidant status
NS	Non-significant
OS	Oxidative stress
